# Identification of Key lncRNAs Associated With Atherosclerosis Progression Based on Public Datasets

**DOI:** 10.3389/fgene.2019.00123

**Published:** 2019-02-28

**Authors:** Chuan-hui Wang, Hui-hua Shi, Lin-hui Chen, Xiao-li Li, Guo-liang Cao, Xiao-feng Hu

**Affiliations:** ^1^Department of Geriatrics, Shanghai Ninth People’s Hospital, Shanghai Jiao Tong University School of Medicine, Shanghai, China; ^2^Department of Neurology, Zhejiang Hospital, Zhejiang University, Hangzhou, China; ^3^Department of Cardiology, Shanghai Chest Hospital, Shanghai Jiao Tong University, Shanghai, China

**Keywords:** long non-coding RNA, atherosclerosis, WGCNA analysis, co-expression analysis, biomarker

## Abstract

Atherosclerosis is one of the most common type of cardiovascular disease and the prime cause of mortality in the aging population worldwide. However, the detail mechanisms and special biomarkers of atherosclerosis remain to be further investigated. Lately, long non-coding RNAs (lncRNAs) has attracted much more attention than other types of ncRNAs. In our work, we found and confirmed differently expressed lncRNAs and mRNAs in atherosclerosis by analyzing GSE28829. We performed the weighted gene co-expression network analysis (WGCNA) by analyzing GSE40231 to confirm highly correlated genes. Gene Ontology (GO) analysis were utilized to assess the potential functions of differential expressed lncRNAs in atherosclerosis. Co-expression networks were also constructed to confirm hub lncRNAs in atherosclerosis. A total of 5784 mRNAs and 654 lncRNAs were found to be dysregulated in the progression of atherosclerosis. A total of 15 lncRNA-mRNA co-expression modules were identified in this study based on WGCNA analysis. Moreover, a few lncRNAs, such as ZFAS1, LOC100506730, LOC100506691, DOCK9-AS2, RP11-6I2.3, LOC100130219, were confirmed as important lncRNAs in atherosclerosis. Taken together, bioinformatics analysis revealed these lncRNAs were involved in regulating the leukotriene biosynthetic process, gene expression, actin filament organization, t-circle formation, antigen processing, and presentation, interferon-gamma-mediated signaling pathway, and activation of GTPase activity. We believed that this study would provide potential novel therapeutic and prognostic targets for atherosclerosis.

## Introduction

Atherosclerosis is characterized by intima-media thickness (IMT) in the middle membrane of cervical artery and the formation of atherosclerotic plaque (Sluimer and Daemen, 2009). Atherosclerosis is one of the most common types of cardiovascular disease and the prime cause of mortality in the aging population worldwide (Sarnak et al., 2003; Mannino and Buist, 2007). Although the previous studies that indicated Immune system responses and inflammation responses were involved in the progression of atherosclerosis, the detail mechanisms, and special biomarkers of atherosclerosis remained to be further investigated.

Previous studies have revealed that non-coding RNAs, such as miRNAs, lncRNAs, and circRNAs, played important regulatory roles in human diseases. For instance, miRNAs were a type of post-transcriptional regulators involved in mRNAs degradation or translation blocking (Chekulaeva and Filipowicz, 2009; Huntzinger and Izaurralde, 2011). Lately, lncRNAs have attracted much more attention than other types of ncRNAs. lncRNAs were a type of ncRNAs with more than 200 bps and observed to be dysregulated in human diseases, including cancers, diabetes, neurodegenerative disease, and cardiovascular diseases (Zamore and Haley, 2005; Hauptman and Glavač, 2013; Zhang et al., 2013). lncRNAs played crucial roles in regulating genome epigenetic modification, RNA splicing, protein translation and mRNA decay. For instance, XIST was a well-known lncRNAs involved in X chromosome inactivation (McHugh et al., 2015). Of note, emerging studies indicated lncRNAs could serve as a type of novel biomarkers for diseases. For example, PCA3 was a potential prognostic marker of prostate cancer, which was more sensitive than the most widely used biomarker, prostate specific antigen (PSA) (Leyten et al., 2014).

In the past decades, a few lncRNAs had been found to be involved in the progression and prognosis of atherosclerosis. For example, Shen and She (2018) reported rs145204276 in the promoter region of GAS5 was associated with the risk of atherosclerosis. Yao et al. (2018) found ENST00113 promotes cell growth and metastasis in atherosclerosis via PI3K/Akt/mTOR pathway. Moreover, H19 was also involved in atherosclerosis through influencing NF-kB and MAPK pathway (Ding et al., 2018). However, there was still lacking system identification of differently expressed lncRNAs in atherosclerosis. Exploring the functions and mechanisms of atherosclerosis related lncRNAs will be useful for the identification of novel biomarkers for this disease.

The Weighted gene co-expression network analysis (WGCNA) method was widely used to identifying key genes involved in human diseases progression. In our work, atherosclerosis related lncRNAs were detected by analyzing GEO datasets GSE28829. Furthermore, we performed the WGCNA to analyze GSE40231 to confirm highly correlated genes. Bioinformatics analysis were also performed to reveal the potential functions of atherosclerosis related lncRNAs. We thought this study will provide novel biomarkers associated with atherosclerosis prognosis and progression.

## Materials and Methods

### Data Sources

The public datasets, GSE28829 and GSE40231, were downloaded from the NCBI Gene Expression Omnibus database. GSE28829 included 13 primary atherosclerotic plaques and 16 advanced atherosclerotic plaques. GSE40231 included 278 atherosclerotic samples from 66 patients. The original data were converted into recognizable format in R, and the preprocess Core package was used for the normalization. Afterward, the limma package of R was used to identify the differentially expressed genes (DEGs) in the progression of atherosclerosis.

### Data Preprocessing

The R software package affy (Gautier et al., 2004) was used to read the microarray data. The robust multiarray business method (Hoffmann et al., 2006) was used for data preprocessing. For the GSE28829 dataset, we identified differently expressed genes using the limma package (Ritchie et al., 2015). The DEG with adjusted *P*-value of less than 0.05 was selected.

### lncRNA Classification Pipeline

In this work, a pipeline was utilized to identify lncRNA expression pattern in atherosclerosis, which was described by Zhang et al., 2012).

### Weighted Gene Co-expression Network Analysis (WGCNA) Analysis

In this study, we conducted WGCNA to predict the potential roles of lncRNAs in atherosclerosis progression. The WGCNA R package was used to evaluate the significance of the two lncRNAs and their module membership. We assessed the weighted co-expression relationship among all dataset subjects in an adjacency matrix using the pairwise Pearson correlation. Following the identification of weighted correlation, the network was presented by Cytoscape 3.4.0.

### Functional Group Analysis

Here, we used GO analysis and KEGG analysis to predict the potential roles of genes by using DAVID system^1^ (Huang et al., 2009).

### Identification of lncRNA-Associated PPI Modules

We applied the analysis of the interaction between lncRNA and protein by utilizing STRING online software was utilized to analyze (Zhang et al., 2016) and the combined score >0.4 was used as the cut-off criterion (Yan et al., 2017). The PPI network was built by utilizing Cytoscape software (Kohl et al., 2011).

## Results

### Identification of Atherosclerosis Progression Related mRNAs and lncRNA

**FIGURE 1:**
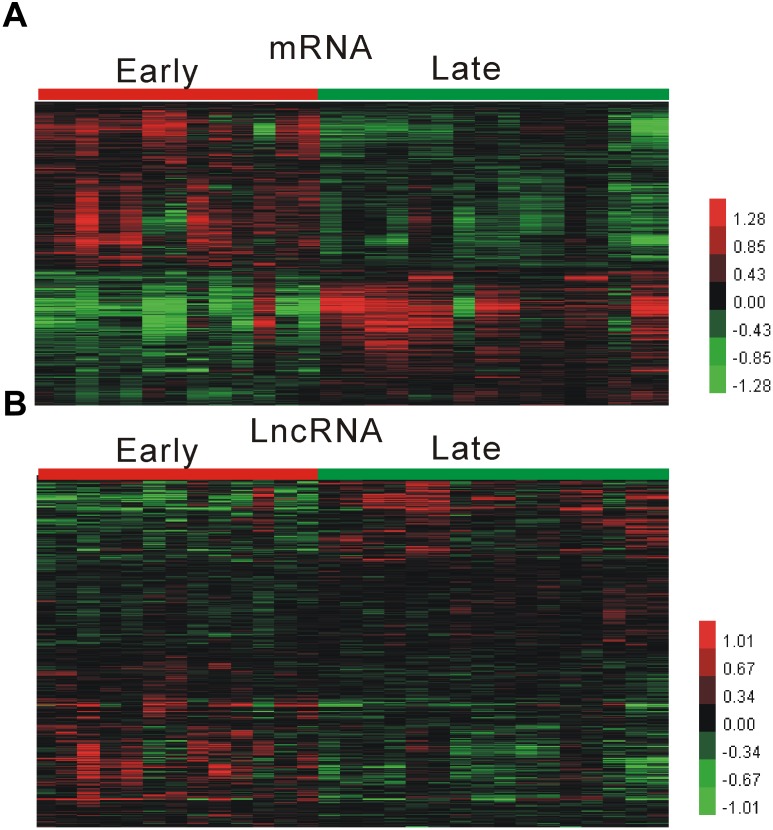
Identification of atherosclerosis progression related mRNAs and lncRNA. **(A,B)** Hierarchical clustering analysis shows differential mRNAs **(A)** and lncRNAs **(B)** expression in advanced atherosclerosis samples compared to early atherosclerosis samples by using GSE28829.

We performed analysis of a public dataset GSE28829 to identify atherosclerosis related mRNAs and lncRNA. GSE28829 was reported by Döring et al. (2012), and contained 13 early and 16 advanced atherosclerosis samples. As shown in Figure 1A, we identified 3542 up-regulated mRNAs and 2487 down-regulated mRNAs in advanced atherosclerosis samples compared to early atherosclerosis samples.

After applying lncRNA classification according to Hägg et al. (2009) report, we identified 356 up-regulated mRNAs and 412 down-regulated lncRNAs in advanced atherosclerosis samples compared to early atherosclerosis samples Figure 1B. However, most of these lncRNA, such as RP11-212P7.2, RP11-498E2.7, RP11-803D5.4, RP11-646J21.6, and RP11-334C17.5, were for the first time observed to be associated with atherosclerosis.

### Construction and Analysis of Gene Co-expression Network

In order to explore the potential functions and mechanisms of these lncRNAs in the progression of atherosclerosis, we conducted WGCNA10 analysis using GSE40231. The network was built by utilizing the WGCNA10.11 package in R software (Langfelder and Horvath, 2008). After identifying the best parameter (β = 4), we applied the WGCNA analysis according to Langfelder et al.’s (2008) reports.

**FIGURE 2:**
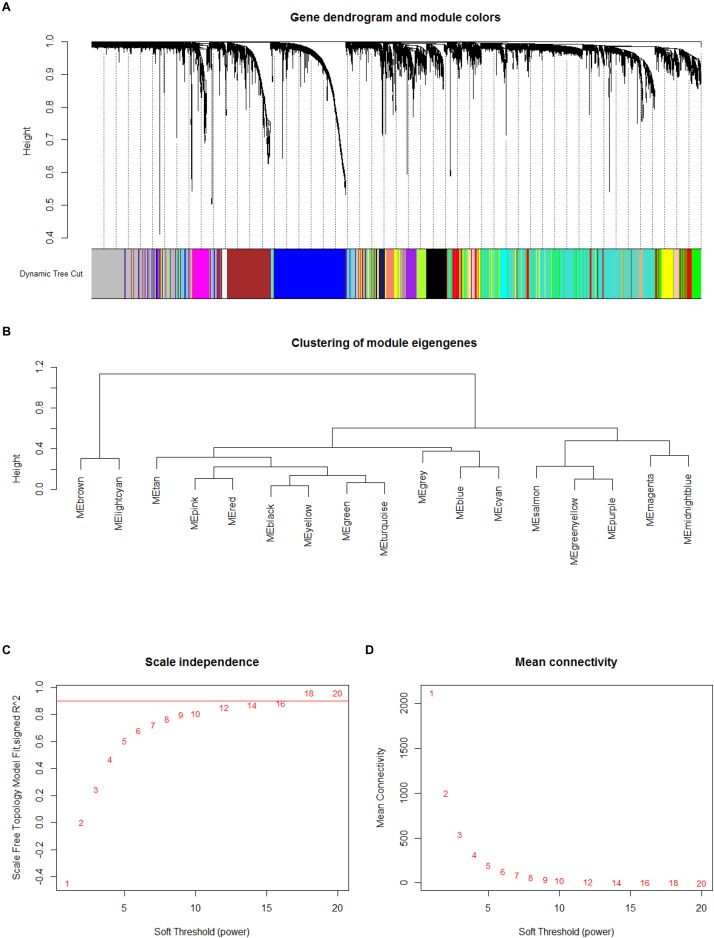
Result of weighted gene correlation network analysis (WGCNA) analysis. **(A,B)** Cluster result and trait heatmap of data samples **(A)** and determination of parameter β of the adjacency function in the WGCNA algorithm **(B)**. **(C)** The scale independence of WGCNA analysis. **(D)** The mean connectivity of WGCNA analysis.

Based on such hypothesis, we acquired 15 gene modules (Figure 2A). We acquired 78 gene modules by using cutreeDynamic in WGCNA package (Langfelder and Horvath, 2008). According to Pidsley et al. (2014) reports, the soft thresholding power five was selected, then, a large minimum module size 10, and a medium sensibility (deep Split = 2) were utilized to segment cluster (Figure 2B). After the Pearson correlation coefficient between modules was calculated, the key network was built (Figure 2C,D). When the absolute value of correlation was greater than 0.45, two modules would be connected.

### Construction of Atherosclerosis Related lncRNA-mRNA Co-expression Networks

**FIGURE 3:**
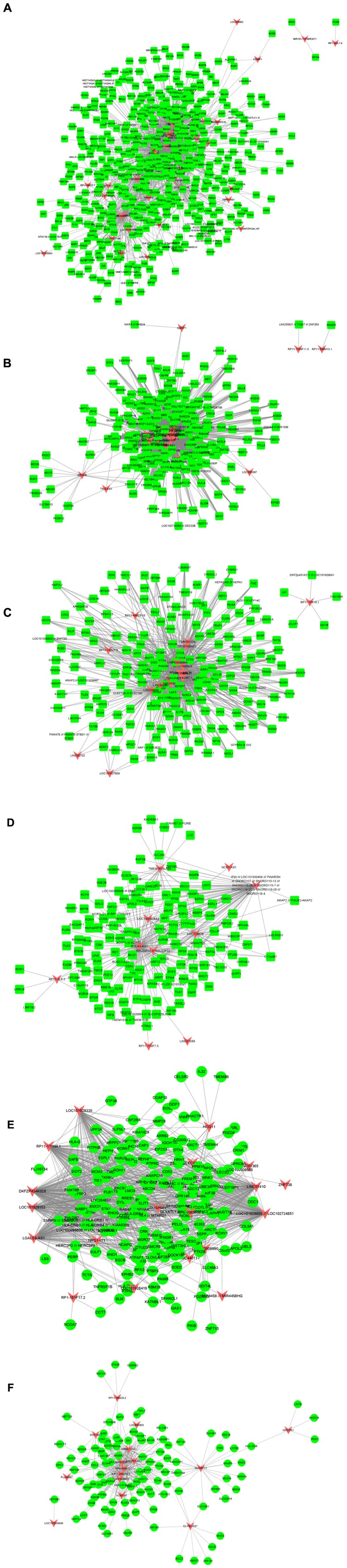
Result of atherosclerosis related lncRNA-mRNA co-expression networks. Construction of module 7–12 lncRNA-mRNA co-expression networks based on WCGNA analysis. **(A–F)** The co-expression networks of module 1 **(A)**, module 2 **(B)**, module 3 **(C)**, module 4 **(D)**, module 5 **(E)**, and module 6 **(F)**.

**FIGURE 4:**
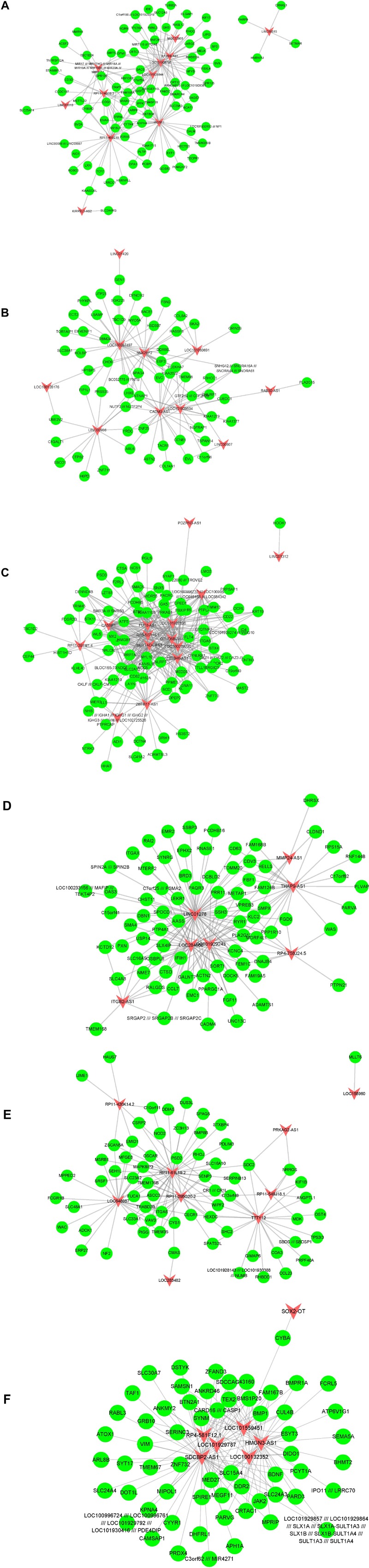
Result of atherosclerosis related lncRNA-mRNA co-expression networks. Construction of module 7–12 lncRNA-mRNA co-expression networks based on WCGNA analysis. **(A–F)** The co-expression networks of module 7 **(A)**, module 8 **(B)**, module 9 **(C)**, module 10 **(D)**, module 11 **(E)**, and module 12 **(F)**.

**FIGURE 5:**
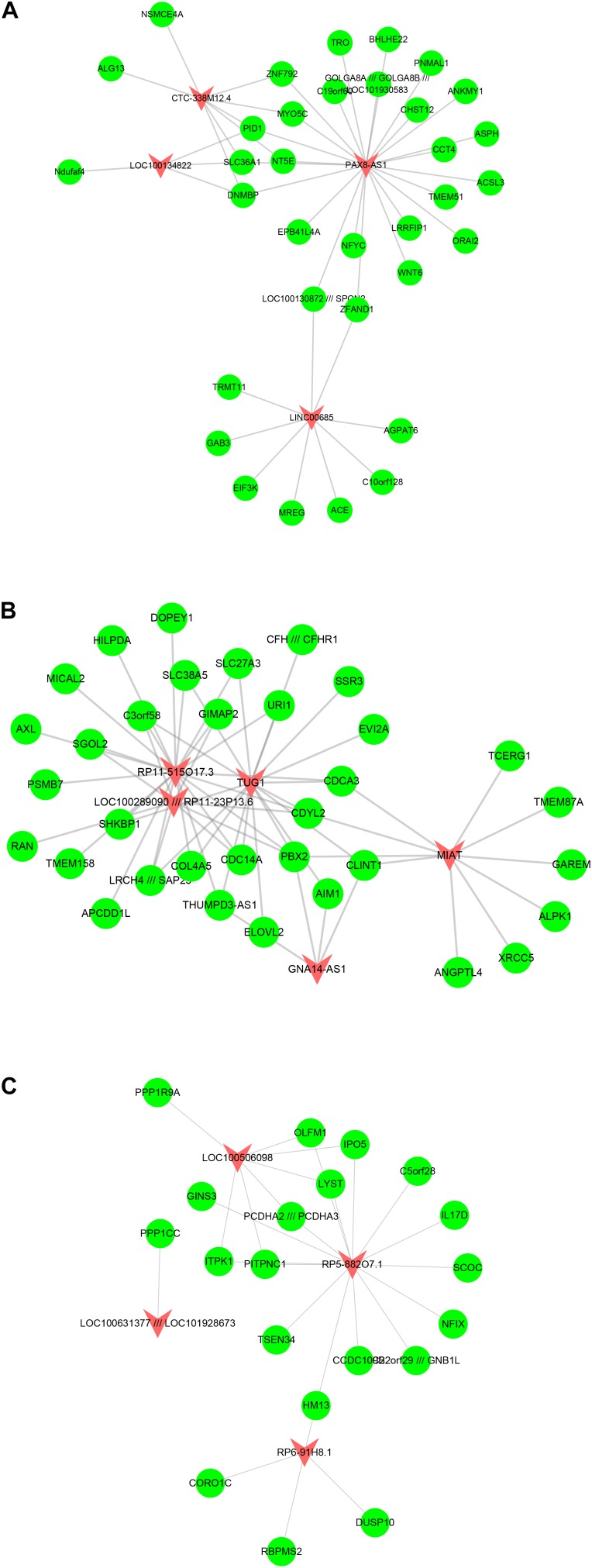
Result of atherosclerosis related lncRNA-mRNA co-expression networks. Construction of module 13–15 lncRNA-mRNA co-expression networks based on WCGNA analysis. The co-expression networks of module 13 **(A)**, module 14 **(B)**, and module 15 **(C)**.

Furthermore, we built atherosclerosis associated lncRNA-mRNA co-expression networks by the Pearson correlation coefficient of lncRNA-mRNA pairs in 15 gene modules based on WCGNA analysis. lncRNA-mRNA pairs with |*R*| > 0.65 were selected for co-expression networks construction. Our results revealed that module 1 related network consisted of 36 lncRNAs and 520 DEGs, module 2 related network consisted of 22 lncRNAs and 229 DEGs, module 3 related network consisted of 17 lncRNAs and 195 DEGs, module 4 related network consisted of 10 lncRNAs and 143 DEGs, module 5 related network consisted of 19 lncRNAs and 175 DEGs, module 6 related network consisted of 15 lncRNAs and 104 DEGs (Figure 3), module 7 related network consisted of 11 lncRNAs and 89 DEGs, module 8 related network consisted of 10 lncRNAs and 61 DEGs, module 9 related network consisted of 14 lncRNAs and 88 DEGs, module 10 related network consisted of 7 lncRNAs and 79 DEGs, module 11 related network consisted of 9 lncRNAs and 66 DEGs, module 12 related network consisted of 7 lncRNAs and 60 DEGs (Figure 4), module 13 related network consisted of 4 lncRNAs and 34 DEGs, module 14 related network consisted of 5 lncRNAs and 34 DEGs, module 15 related network consisted of 4 lncRNAs and 20 DEGs (Figure 5).

A few lncRNAs, such as ZFAS1 (degree = 358), LOC100506730 (degree = 183), LOC100506691 (degree = 170), DOCK9-AS2 (degree = 167), RP11-6I2.3 (degree = 166), LOC100130219 (degree = 157), LOC100268168 (degree = 138), DAPK1-IT1 (degree = 130), LOC100507250 (degree = 129), HLA-J (degree = 128), and LOC102723845 (degree = 121), were identified as key regulators in this network.

### Function Annotation of Atherosclerosis Related lncRNAs

**FIGURE 6:**
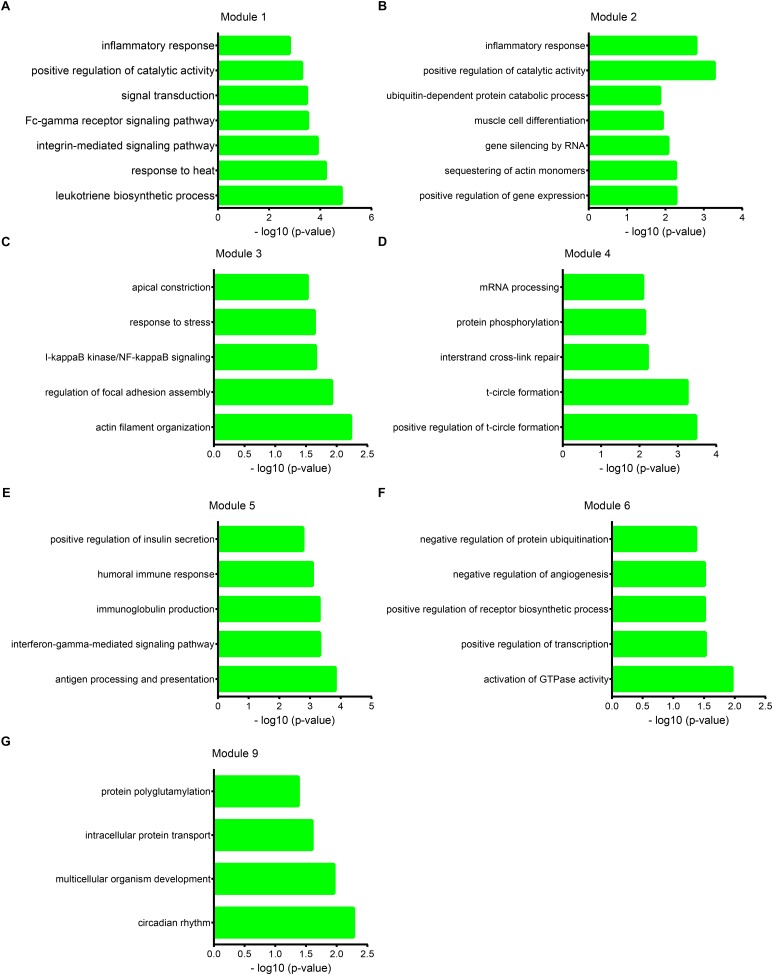
Function annotation of atherosclerosis related lncRNAs. Gene Ontology (GO) analysis shows lncRNAs in module 1 **(A)**, module 2 **(B)**, module 3 **(C)**, module 4 **(D)**, module 5 **(E)**, module 6 **(F)**, and module 9 **(G)** regulate multiple biological processes.

Furthermore, we performed bioinformatics analysis for atherosclerosis related lncRNAs using DAVID system. Our results showed lncRNAs in module 1 were involved in regulating leukotriene biosynthetic process, response to heat, integrin-mediated signaling pathway, Fc-gamma receptor signaling pathway involved in phagocytosis, signal transduction, positive regulation of catalytic activity, and inflammatory response. lncRNAs in module 2 were involved in regulating positive regulation of gene expression, sequestering of actin monomers, gene silencing by RNA, muscle cell differentiation, and ubiquitin-dependent protein catabolic process. lncRNAs in module 3 were involved in regulating actin filament organization, regulation of focal adhesion assembly, I-kappaB kinase/NF-kappaB signaling, response to stress, and apical constriction. lncRNAs in module 4 were involved in regulating positive regulation of t-circle formation, t-circle formation, interstrand cross-link repair, protein phosphorylation, and mRNA processing Figure 6.

lncRNAs in module 5 were involved in regulating antigen processing and presentation, interferon-gamma-mediated signaling pathway, immunoglobulin production, humoral immune response, and positive regulation of insulin secretion. lncRNAs in module 6 were involved in regulating activation of GTPase activity, positive regulation of transcription, positive regulation of receptor biosynthetic process, negative regulation of angiogenesis, and negative regulation of protein ubiquitination. lncRNAs in module 7 were involved in positive regulation of DNA topoisomerase activity, and ossification. lncRNAs in module 8 were involved in positive regulation of protein import into nucleus. lncRNAs in module 9 were involved in circadian rhythm, multicellular organism development, intracellular protein transport, protein polyglutamylation. lncRNAs in module 10 were involved in response to virus, positive regulation of GTPase activity, cellular response to caffeine. lncRNAs in module 12 were involved in cell cycle, positive regulation of t-circle formation, t-circle formation Figure 6.

## Discussion

Atherosclerosis had been the prime cause of mortality in the ageing population worldwide. However, the detail mechanisms underlying atherosclerosis progression and accurate biomarker for the prognosis of atherosclerosis remained to be investigated. In our work, we aimed to confirm atherosclerosis related lncRNAs and mRNAs using GSE28829 and GSE40231. Totally, 5784 mRNAs and 654 lncRNAs were identified to be dysregulated in the progression of atherosclerosis. WGCNA was performed to identify highly correlated lncRNAs and mRNAs. Moreover, co-expression network and bioinformatics analysis were used to find the potential functions of lncRNAs in atherosclerosis.

lncRNAs played crucial roles in human diseases via binding to DNA, proteins and RNA molecules. Recently, a few lncRNAs, such as GAS5 (Chen et al., 2017), NEAT1 (Jian et al., 2016) and MALAT1 were reported to be associated with regulating atherosclerosis progression and prognosis. For example, interactions among MALAT1 (Han et al., 2018), NEAT1, and key immune effector molecules could regulate the development of atherosclerosis. However, still lacking was a systematic identification of differentially expressed lncRNAs in atherosclerosis. In this study, we identified 356 up-regulated mRNAs and 412 down-regulated lncRNAs in advanced compared to early atherosclerosis samples. Among these lncRNAs, MBNL1-AS1, HAND2-AS1, and RP11-999E24.3 were most down-regulated and PSMB8-AS1, LINC01094, and RP11-389C8.2 were most up-regulated lncRNAs in advanced atherosclerosis patients. Interestingly, we observed several well-known lncRNAs, such as TUG1, PCA3, and HOTAIR, which were also involved in regulating atherosclerosis progression. A previous study showed TUG1 knockdown could ameliorate atherosclerosis via inducing FGF1 expression (Zhang et al., 2018). Moreover, TUG1 was reported to be an oncogene in various types of human cancers, such as colorectal cancer, ovarian cancer, and gastric cancer (Huarte, 2015). PCA3 was a novel potential biomarker for prostate cancer (Leyten et al., 2014). HOTAIR is abnormally expressed in cancers and involved in regulating cancer proliferation, cell cycle and apoptosis (Esteller, 2011). This study together with previous studies demonstrated lncRNAs also played key roles in the progression of Atherosclerosis.

One of the biggest challenges in exploring the functions of lncRNAs in human diseases was that lncRNAs could not be used to perform GO and KEGG analysis. In previous studies, many groups conducted bioinformatics analysis for lncRNAs using their co-expressing genes. For instance, Feng et al. (2018) identified and predicted the functions of implantation failure related lncRNAs by constructing the lncRNA-mRNA co-expression network. In order to study the potential functions of atherosclerosis-related lncRNAs, we performed WGCNA analysis. A total of 15 lncRNA-mRNA co-expression modules were identified in this study. A few lncRNAs, such as ZFAS1, LOC100506730, LOC100506691, DOCK9-AS2, RP11-6I2.3, LOC100130219, LOC100268168, DAPK1-IT1, LOC100507250, and LOC102723845, were confirmed as important lncRNAs due to that they co-expressed with more than 100 different mRNAs in Atherosclerosis. Besides ZFAS1, the roles of these lncRNAs remained unknown. Here, we found ZFAS1 played the most important roles in this network though co-expressing with 358 mRNAs. ZFAS1 was dysregulated in breast cancer, gastric cancer, and colorectal cancer, and played as an oncogene in cancer progression though promoting cancer metastasis, growth and EMT. Furthermore, we performed bioinformatics analysis and observed these dysregulated lncRNAs were significantly associated with the regulation of leukotriene biosynthetic process, gene expression, actin filament organization, t-circle formation, antigen processing, and presentation, interferon-gamma-mediated signaling pathway, and activation of GTPase activity. Of note, we observed lncRNAs in module 5, such as RP11-171N4.1, DKFZP434K028, LOC101929153, LGALS8-AS1, and LINC01410, were significantly involved in regulating immune system responses and inflammation responses, which had been reported to be key regulators in atherosclerosis.

We should point out that there were several limitations included in this study. First, the expression levels of key lncRNAs in atherosclerosis was not validated using clinical samples. Second, the detail of molecular functions of key lncRNAs in the progression of atherosclerosis had not been investigated. Therefore, the further validation and function investigation will still require further study.

## Conclusion

In conclusion, we identified a total of 275 lncRNAs were found to be dysregulated in the progression of atherosclerosis. WGCNA was performed to identify highly correlated lncRNAs and mRNAs. Moreover, ZFAS1, LOC100506730, LOC100506691, DOCK9-AS2, RP11-6I2.3, and LOC100130219 were identified as key lncRNAs in atherosclerosis. Bioinformatics analysis revealed these lncRNAs were involved in regulating the leukotriene biosynthetic process, gene expression, actin filament organization, t-circle formation, antigen processing, and presentation, interferon-gamma-mediated signaling pathway, and activation of GTPase activity. This research would provide potential novel therapeutic and prognostic targets for atherosclerosis.

## Author Contributions

C-hW, H-hS, G-lC, and X-fH conceived and designed the study. C-hW, H-hS, L-hC, and X-lL developed the methodology. L-hC and X-lL collected the sample. C-hW, H-hS, L-hC, and X-lL analyzed and interpreted the data. C-hW, H-hS, L-hC, X-lL, G-lC, and X-fH wrote, reviewed, and/or revised the manuscript.

## Conflict of Interest Statement

The authors declare that the research was conducted in the absence of any commercial or financial relationships that could be construed as a potential conflict of interest.

## Footnotes

1 https://david.ncifcrf.gov/
